# A Member of One of the Learned Professions

**Published:** 1897-06

**Authors:** 


### A Member of One of the Learned Professions.

A pretty state of affairs was brought to light in Bay City, Mich., recently. It seems that there has flourished there for some years a practicing physician by the name of O. Barber, who is also a retail druggist. Recently Dr. Barber had a case of diphtheria which he failed to report. For this oversight he was overhauled by the Health Board, and the matter brought into court. To the amazement of that body and probably to the horror of his patients, he gave testimony to the fact that he could neither read nor write. In some mysterious, miraculous way, he has been able to make a great big "bluff" all these years. He is a Canadian by birth, and simply came over to the States and called himself "Dr." Barber. He was running the drug sore when the Michigan State Board came into existence and it granted him a certificate as a registered pharmacist, without an examination—as it did everybody else who happened to be in a drug-store at that time. How he ever managed no one knows, but it is presumed that most of his business consisted in putting up his own medicines. The exposure of this man has precipitated a war in Bay City against quacks and quackery, and the result will probably be very wholesome.—*Pharmaceutical Era.*

